# Modulation of the intestinal microbiota: impact on productive performance and intestinal health in broiler breeder hens

**DOI:** 10.1016/j.psj.2026.107703

**Published:** 2026-08-26

**Authors:** Dauísia Corsino de Souza, Roberto Carlos de Freitas, Manoel Garcia Neto, Jumara Coelho Ticiano, Raphael Lucio Andreatti, Adriano Sakai Okamoto

**Affiliations:** a São Paulo State University (UNESP), School of Veterinary Medicine and Animal Science, Botucatu, SP, 18618-681, Brazil; b Department of Animal Science, São Paulo State University, Araçatuba, SP, 16050-680, Brazil; c School of Animal Science and Food Engineering (FZEA), University of São Paulo (USP), Pirassununga, SP, Brazil

**Keywords:** Microbial supplementation, 16S rRNA sequencing, Beta diversity, Gastrointestinal homeostasis

## Abstract

This study evaluated the effects of different administration protocols of an undefined microbial culture (Normal Avian Gut Flora — NAGF) on production performance metrics, intestinal health, and cecal microbiota composition in Cobb 500 broiler breeder hens during the early production phase (24 to 32 weeks of age). A total of 48,000 broiler breeder hens were allocated across four commercial units, including an unsupplemented control group and three treatment groups receiving NAGF via drinking water at different application frequencies: weekly (Group 1), biweekly (Group 2), and every 21 days (Group 3). Flock performance metrics (egg production and mortality) and macroscopic clinical health scores were analyzed using Generalized Linear Mixed Models (GLMM) for longitudinal repeated measures. Cecal microbiota composition was evaluated at 32 weeks from 20 biological sample pools (n=5 pools/group, derived from 100 hens) via 16S rRNA gene amplicon sequencing. To distinguish statistical significance from practical relevance in this large-scale field trial, biological response magnitudes were prioritized using effect size estimators [Exp(B)]. Administration protocols significantly influenced flock survival and lay performance. The biweekly protocol (Group 2) provided optimal protective efficiency, exhibiting a lower estimated mortality rate than the Control group [Exp(B)=0.753; P=0.012] and superior laying persistence relative to controls. Conversely, extending the administration window to 21 days (Group 3) was counterproductive, resulting in a higher estimated mortality rate [Exp(B)=1.258; P=0.042]. Treated groups exhibited numerical reductions in intestinal gas accumulation and macroscopic lesion scores, although the overall differences were not statistically significant (P > 0.05). Differential abundance analysis using ANCOM-BC confirmed targeted taxonomic shifts, particularly the depletion of potential opportunistic lineages such as Massilistercora and taxa within the order Clostridiales. Collectively, these findings demonstrate that NAGF efficacy is strictly frequency-dependent: biweekly administration optimizes intestinal integrity and flock survival, whereas an extended 21-day interval proves ineffective and should be avoided in commercial health management programs.

## Introduction

Broiler breeder hens play a pivotal role in the sustainability of the poultry production chain, directly influencing reproductive efficiency, chick quality, and overall flock performance (Livingston et al., 2020). However, enteric disorders and impaired gastrointestinal health remain major limiting factors in commercial breeder production, negatively impacting egg laying rates, feed conversion, and flock survival (Rhayat et al., 2019).

Subclinical enteritis and intestinal mucosal disruptions can alter gastrointestinal function, impairing nutrient absorption and compromising intestinal barrier integrity, which may increase susceptibility to pathogen colonization (Zanu et al., 2020). Historically, antimicrobial growth promoters were extensively utilized to mitigate enteric disease pressure and stabilize gut function. However, regulatory restrictions-most notably those implemented across the European Union in 2003-and global public health concerns regarding antimicrobial resistance led to strict limitations on non-therapeutic antibiotic use (EC, 2003). Consequently, research has intensified toward sustainable nutritional and biological strategies capable of supporting gut health, maintaining microbial balance, and protecting flock performance without relying on antimicrobials (Pessôa et al., 2012; Abd El-Hack et al., 2022).

Among these alternatives, undefined competitive exclusion cultures, consisting of complex mixtures of normal avian gut microbiota, have been investigated as a strategy to promote early intestinal colonization and limit the establishment of enteric pathogens (Hofacre et al., 2019). By occupying ecological niches and producing short-chain fatty acids, these complex microbial communities contribute to a stable intestinal ecosystem associated with preserved mucosal integrity and improved flock health (Jha et al., 2020; Dong et al., 2024).

The application of high-throughput sequencing technologies, particularly 16S rRNA gene amplicon sequencing, has expanded the characterization of cecal microbial communities at the taxonomic level, enabling the assessment of microbial diversity, relative taxonomic abundance, community structure, and compositional shifts in response to dietary and microbial interventions (Aruwa et al., 2021; Wang et al., 2022). Despite growing interest in microbial cultures, significant knowledge gaps persist regarding the optimal administration frequency required to maintain long-term competitive exclusion under commercial field conditions. In large-scale operations, dosing schedules often vary widely, making it essential to determine whether repeated or intermittent administration protocols provide optimal microbial stability and performance benefits.

Therefore, this study aimed to evaluate the effects of different administration frequencies of an undefined competitive exclusion culture (NAGF) on cecal microbiota composition, intestinal clinical parameters, and production performance metrics in Cobb 500 broiler breeder hens during the early production phase (24 to 32 weeks of age) using 16S rRNA gene sequencing. We hypothesized that varying NAGF administration intervals would differentially modulate cecal microbial community structure, intestinal gross health parameters, and longitudinal flock performance under commercial production conditions.

## Materials and methods

### Ethics statement

All procedures involving animals were conducted in accordance with established ethical principles and were approved by the Animal Ethics Committee of the School of Veterinary Medicine and Animal Science, São Paulo State University (UNESP), under protocol number 000023/2023.

### Experimental design

#### Experimental design, birds, and housing

The experiment was conducted at a commercial broiler breeder facility located in Itaí, São Paulo, Brazil. A total of 48,000 Cobb 500 broiler breeder hens were evaluated under commercial field conditions between 24 and 32 weeks of age (an 8-week evaluation period). The flock was allocated across four experimental treatment groups, with each group housed in an independent commercial production unit (house) containing approximately 12,000 birds.

#### Microbial culture characterization and administration protocols

The evaluated product was a commercial undefined competitive exclusion culture derived from the Normal Avian Gut Flora (NAGF; **Colostrum® LÍQUIDO**, Code 9005, Batch No. 005/23, Biocamp Laboratório Pesquisa e Desenvolvimento Ltda., Campinas, São Paulo, Brazil). The product is a liquid pharmaceutical form supplied in 50 mL bottles (52mL actual measured volume). According to the manufacturer’s release specifications and official Certificate of Analysis, the guaranteed minimum levels and actual batch test results for active constituents and quality parameters were as follows:

• **Total Anaerobic Bacteria:** Guaranteed minimum of $1.0\;\times 10^{6}\;\text{CFU}/\text{mL}$; actual batch result obtained was $1.0\;\times 10^{8}\;\text{CFU}/\text{mL}$.
• **Lactic Acid-Producing Bacteria:** Guaranteed minimum of $1.0\;\times 10^{6}\;\text{CFU}/\text{mL}$; actual batch result obtained was $6.5\;\times 10^{6}\;\text{CFU}/\text{mL}$.
• **Physical/Chemical Properties:** Light yellow liquid appearance (pH=5.66; specification range 5.0±1.0).
• **Microbiological Safety Standards:** Certified absent of molds, *Pseudomonas* spp., *Salmonella* spp., and *Staphylococcus aureus*. Furthermore, the product tested negative via Polymerase Chain Reaction (PCR) for both *Salmonella* spp. and *Pasteurella multocida*.

The product was stored under refrigeration at $2^{\circ }C$ to $8^{\circ }C$ in accordance with the manufacturer’s instructions and reconstituted in clean, unchlorinated drinking water immediately prior to administration. Supplemental treatments evaluated four distinct administration schedules via drinking water:

• **Control Group:** Received no supplementation and was maintained exclusively on the basal diet and unsupplemented drinking water throughout the 8-week experimental period (0 total exposure days; 0mL product/1,000 birds).
• **Group 1 (Weekly):** Received NAGF in drinking water for 3 consecutive days every 7 days (8 administration cycles; 24 total days of exposure; cumulative dose of 240mL product/1,000 birds).
• **Group 2 (Biweekly):** Received NAGF in drinking water for 3 consecutive days every 15 days (4 administration cycles; 12 total days of exposure; cumulative dose of 120mL product/1,000 birds).
• **Group 3 (21-Day Interval):** Received NAGF in drinking water for 3 consecutive days every 21 days (3 administration cycles; 9 total days of exposure; cumulative dose of 90mL product/1,000 birds).

During active administration days, NAGF was applied at a standardized daily dose of 10mL per 1,000 birds during the first morning water consumption window (approximately an 8-hour intake window, based on an average flock consumption of 350mL/bird/day). Per commercial house (12,000 birds), 120mL of reconstituted product was administered daily (dissolved equally across two 2,000-L header tanks at 60mL/tank, equivalent to 50 mL of diluted tank mixture per 5,000 birds) to guarantee uniform flock exposure. Because the single-dose concentration was held constant across treatments, experimental groups deliberately differed in application frequency and total cumulative exposure over the production phase.

#### Statistical Framework and Pseudoreplication Controls

To maintain analytical rigor and prevent statistical pseudoreplication under commercial field conditions:

• **Microbiome Analysis:** Cecal digesta sampling for 16S rRNA gene amplicon sequencing was performed at 32 weeks of age on 100 birds (25 birds/treatment group). To ensure statistical independence at the biological sample level, digesta from 5 individual birds within the same production house were homogenized into a single biological pool, yielding 20 independent biological pools (n=5 biological pools per group). These pools served as the statistical units for alpha diversity, beta diversity distance matrices (PERMANOVA with 999 permutations), and differential abundance modeling (ANCOM-BC).
• **Performance Metrics and Clinical Health:** Flock performance indicators (egg production, mortality rate) and macroscopic intestinal clinical scores were collected weekly. Parameters were analyzed using Generalized Linear Mixed Models (GLMM) for repeated measures over time, incorporating time points and nested barn-level clustering structures as random effects.

#### Performance metrics evaluation

Primary flock performance parameters were monitored weekly throughout the 8-week experimental period (from 24 to 32 weeks of age), including weekly mortality rates and egg production metrics.

Mortality was recorded daily within each commercial production unit (house) and summarized weekly as a percentage of initial flock size. Weekly mortality served as a direct quantitative indicator of bird viability, general clinical resilience, and overall flock health during the physiological strain of peak lay.

Egg production was recorded daily per production unit and expressed as weekly average hen-housed egg production rate (%) and hen-day egg production rate.

To evaluate the longitudinal dynamics of the administration protocols on flock performance, data were analyzed using Generalized Linear Mixed Models (GLMM) for repeated measures over time, incorporating nested barn-level clustering structures as random effects. Weekly mortality rates were modeled assuming a Gamma distribution with a log link function (or Poisson/binomial structure where appropriate) to account for asymmetric continuous response data, whereas weekly egg production rates (%) were analyzed using Beta regression with a logit link function following transformation to the (0,1) open interval to accommodate proportional data. Bird age (weeks) was included as a continuous covariate with polynomial terms (quadratic and cubic) to model non-linear longitudinal trajectories.

Given the large scale of this commercial trial (48,000 birds), model evaluation prioritized effect size estimators to distinguish true biological and practical relevance from statistical overpower. Treatment effects and mortality rate ratios were presented as exponentiated coefficients [Exp(B)], relative rate reductions, and estimated marginal means with statistical significance set at P≤0.05.

### Intestinal microbiota analysis

Cecal content samples were collected at **32 weeks of age,** from 100 birds (25 birds per treatment group). To prevent statistical pseudoreplication, equal amounts of cecal digesta from 5 individual birds within the same treatment house were homogenized to construct 20 independent biological pools (n=5 biological pools per group).

Total genomic DNA extraction, library preparation, and sequencing were conducted at the Institute for Biotechnology (IBTEC, São Paulo State University — UNESP, Botucatu, Brazil). Libraries were prepared following Illumina’s *16S Metagenomic Sequencing Library Preparation* protocol. Amplicons targeting the hypervariable regions of the bacterial 16S rRNA gene were generated and sequenced on an Illumina NextSeq platform, targeting an average bacterial genome coverage depth of 50 × .

Sequence processing and bioinformatic analyses were executed within the QIIME 2 environment. Raw sequence data and QIIME 2 visualization files (.qzv) were deposited and made accessible in a dedicated public repository (https://drive.google.com/drive/folders/1r2PCL7b5m2arkrs-hboPxFLeXcfPDY2S?usp=sharing).

Alpha diversity was evaluated using Observed Features, Shannon Index, Faith’s Phylogenetic Diversity, and Pielou’s Evenness metrics. Beta diversity community structure was assessed using distance-based matrices, including Bray–Curtis dissimilarities, Jaccard distances, unweighted UniFrac, and weighted UniFrac distances. Pairwise Permutational Multivariate Analysis of Variance (PERMANOVA) with 999 permutations was used to test for differences in beta diversity structure among groups.

Differential abundance of bacterial taxa across Order (level 4), Family (level 5), and Genus (level 6) were determined using the Analysis of Compositions of Microbiomes with Bias Correction (ANCOM-BC) method at a significance level of α=0.05.

### Statistical analysis

All performance and intestinal clinical data were analyzed considering the longitudinal nature of the experiment and the temporal evolution of indicators throughout the 8-week production period (24 to 32 weeks of age). Statistical modeling for flock performance metrics and macroscopic gut clinical evaluations was performed using Generalized Linear Mixed Models (GLMM) to account for repeated measures over time and random clustering effects associated with commercial production units.

For flock performance parameters, weekly mortality rates were modeled using GLMM assuming a Gamma distribution with a log link function (or Poisson/binomial structure where appropriate), permitting the proper modeling of continuous asymmetric data and the interpretation of treatment effects via exponentiated coefficients [Exp(B)]. Weekly egg production rates (%) were analyzed using Beta regression with a logit link function following transformation to the (0,1) open interval to account for proportional data. Models included fixed effects for treatment group, bird age (weeks), and polynomial terms (quadratic/cubic) to capture non-linear temporal dynamics.

Intestinal clinical indicators and macroscopic intestinal lesion scores (evaluated via an ordinal clinical scoring system: Normal, Score 1, Score 2, and Score 3) were modeled using GLMM with a Negative Binomial distribution and log link function to address count data structure and potential overdispersion. Fixed effects in the models comprised treatment group, bird age, specific clinical parameter, and their higher-order interactions. Treatment effects were expressed as incidence rate ratios using exponentiated coefficients [Exp(B)]. The integrated intestinal condition index (combining mucus and gas presence) was analyzed using GLMM with a Gaussian distribution and identity link function to evaluate intestinal stability over time.

Given the large-scale nature of this commercial field trial (48,000 birds), reliance solely on hypothesis testing (P-values) introduces the risk of statistical "overpowering," wherein trivial biological differences reach formal statistical significance (P<0.05) due to near-zero standard errors. To distinguish statistical significance from biological and practical relevance in flock performance metrics and clinical scores, effect sizes—expressed as exponentiated coefficients [Exp(B)], rate ratios, and relative rate percentages—were prioritized to quantify the magnitude of treatment impacts. Evaluating effect sizes ensures standardized comparability across production cycles, facilitates cost-benefit decision-making for commercial application, and provides a robust foundation for future meta-analyses without sample-size distortion.

For cecal microbiota analyses, high throughput 16S rRNA sequencing data were processed independently within the QIIME 2 environment at the biological sample pool level (n=5 pools per treatment group; 20 total pools). Differences in beta diversity community structure across treatment groups were evaluated using Pairwise Permutational Multivariate Analysis of Variance (PERMANOVA) with 999 permutations based on Bray–Curtis, Jaccard, unweighted UniFrac, and weighted UniFrac distance matrices. Differential abundance of bacterial taxa across Order (level 4), Family (level 5), and Genus (level 6) was evaluated using Analysis of Compositions of Microbiomes with Bias Correction (ANCOM-BC) with Benjamini–Hochberg false discovery rate (FDR) corrections for multiple testing (α=0.05).

Statistical significance was declared at P≤0.05, with numerical trends discussed at 0.05<P≤0.10.

## Results

### Flock performance metrics

#### Mortality

Mortality rate exhibited a nonlinear pattern throughout the evaluation period (24 to 32 weeks of age), with a progressive increase until approximately 28 weeks of age, followed by stabilization and a slight decline during subsequent weeks (Fig. 1). This pattern reflects a typical physiological response during the early production phase, associated with metabolic adaptation to peak production and inherent health challenges during this stage.

**Fig. 1:**
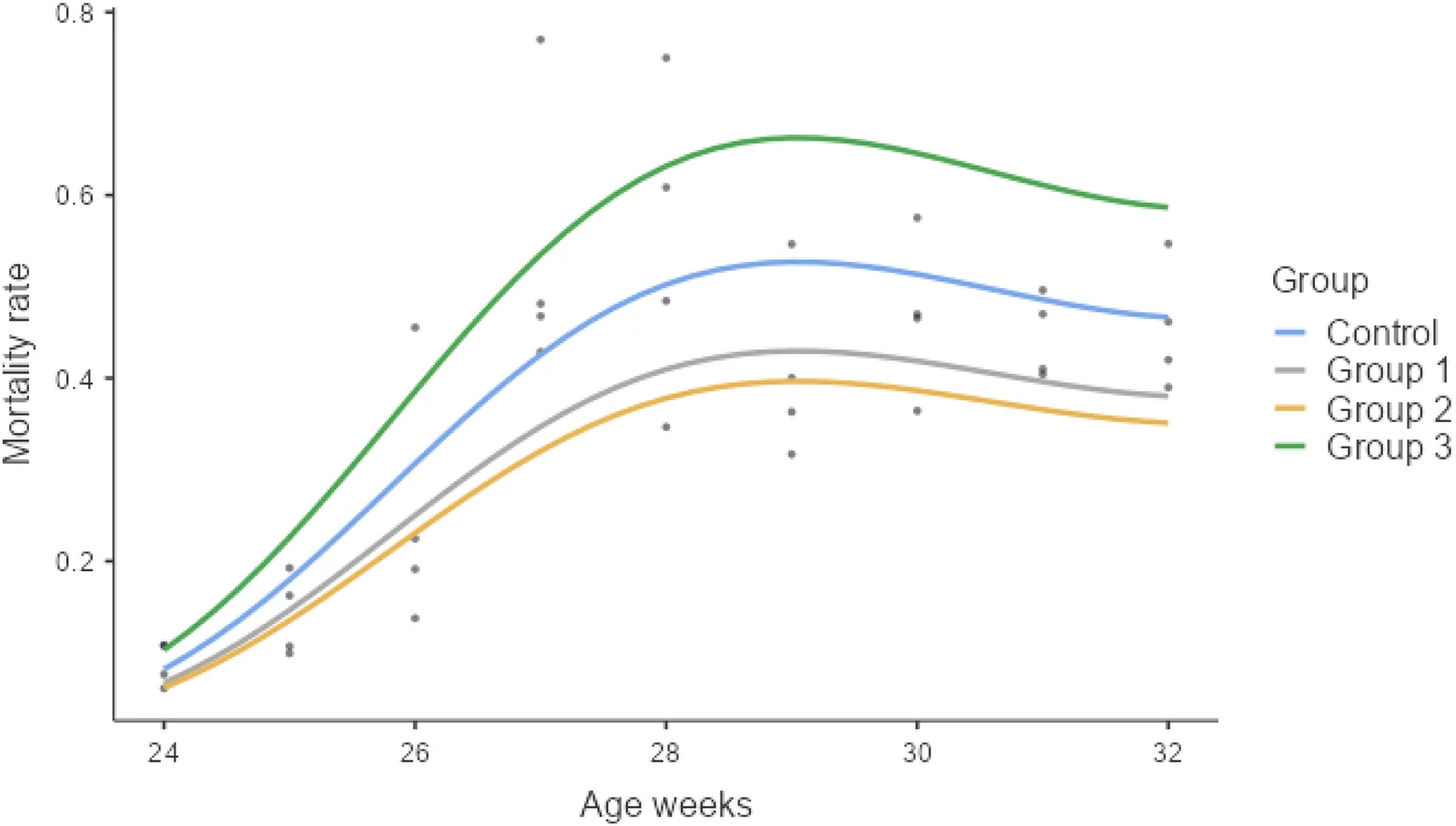
Weekly mortality rate pattern in broiler breeder hens subjected to different microbial administration protocols. Lines represent model fitted curves across age, whereas points represent the observed values.

The Generalized Linear Mixed Model (GLMM) revealed a significant main effect of treatment group on flock mortality ($\chi^{2}=25.14$; df=3; P<0.001), indicating consistent differences among administration protocols. Additionally, bird age had a significant linear effect ($\chi^{2}=7.69$; P=0.0056), as well as significant quadratic ($\chi^{2}=6.38$; P=0.0115) and cubic terms ($\chi^{2}=5.31$; P=0.0212), confirming the nonlinear trajectory of mortality over time.

Estimated marginal means demonstrated distinct differences among experimental groups (Table 1). The highest mortality rate was observed in Group 3 (0.6318%), followed by the unsupplemented Control group (0.5024%), Group 1 (0.4096%), and the lowest rate in Group 2 (0.3781%). These results highlight the superior efficacy of the biweekly administration protocol (Group 2), which achieved the lowest overall mortality rate among all evaluated treatments.

**Table 1:** Estimated marginal means of mortality according to microbial administration protocols.

Group	Mean (%)	SE	Lower (95% CI)	Upper (95% CI)
Control	0.5024	0.0304	0.4439	0.5686
Group 1	0.4096	0.0426	0.3311	0.5068
Group 2	0.3781	0.0393	0.3056	0.4677
Group 3	0.6318	0.0657	0.5107	0.7816

SE = standard error; CI = confidence interval.

Analysis of exponentiated coefficients [Exp(B)] demonstrated a reduction in mortality rate for groups receiving regular microbial administration relative to the Control group. Group 1 exhibited a trending reduction in mortality rate [Exp(B)=0.815; P=0.071], indicating that the Control group had a mortality rate approximately 22.7% higher than the weekly protocol [((1/0.815)−1)×100]. Group 2 showed a significantly more pronounced protective effect [Exp(B)=0.753; P=0.012], with the Control group exhibiting a 32.8% higher mortality rate than the biweekly treatment [((1/0.753)−1)×100]. Conversely, Group 3 (21-day interval) exhibited an increased mortality rate relative to the Control group [Exp(B)=1.258; P=0.042], corresponding to a 25.8% increase in mortality rate [(1.258−1)×100].

Overall, these findings indicate that the frequency of administration directly influences bird survival during the early laying period, with the biweekly protocol (every 15 days) providing the optimal balance for maintaining intestinal stability and physiological resilience under commercial conditions.

#### Egg production

Egg production exhibited a nonlinear pattern throughout the evaluation period, with a progressive increase beginning at 24 weeks of age, reaching peak values at approximately 28 weeks, followed by stabilization during the subsequent weeks (Fig. 2). This pattern reflects the consolidation of the productive phase, characterized by physiological adaptation and increased reproductive efficiency over time.

**Fig. 2:**
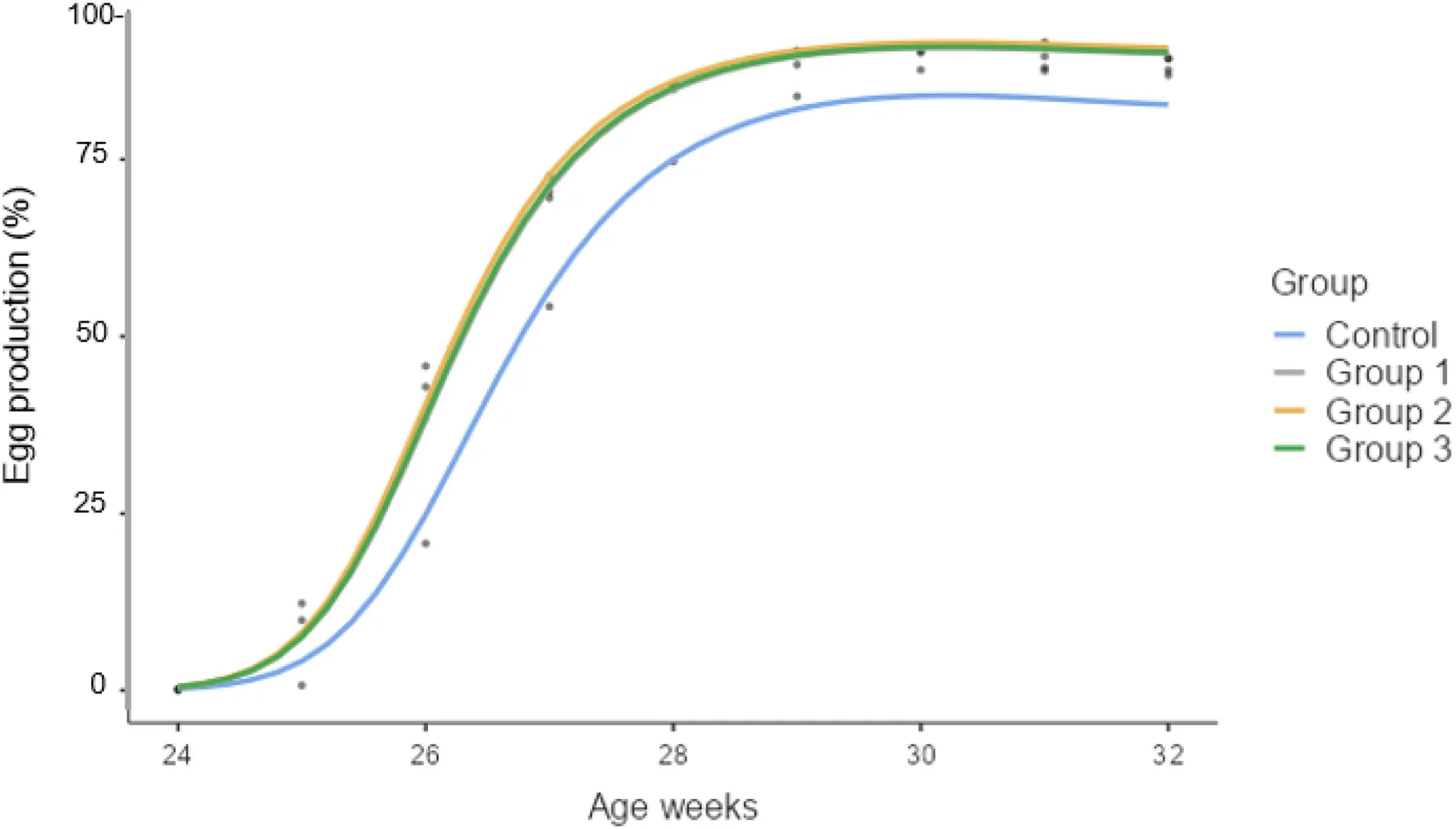
Weekly egg production pattern in broiler breeder hens subjected to different microbial administration protocols. Lines represent model fitted curves across age, whereas points represent the observed values.

The Generalized Linear Model revealed a significant effect of treatment group on egg production (χ² = 57.9; df = 3; p < 0.001), indicating consistent differences among microbial administration protocols. Age also had a significant effect (χ² = 32.2; p < 0.001), as did its quadratic (χ² = 26.5; p < 0.001) and cubic polynomial terms (χ² = 21.9; p < 0.001), confirming a well-defined nonlinear pattern of egg production over time.

Estimated marginal means indicated higher egg production in the treated groups compared with the control group (Table 2), demonstrating a consistent positive effect of microbial supplementation on productive performance. The highest mean value was observed in Group 2 (86.9%), followed by Groups 3 (86.0%) and 1 (85.8%), whereas the control group exhibited the lowest mean production (76.3%).

**Table 2:** Estimated marginal means of egg production according to microbial administration protocols.

Group	Mean (%)	SE	Lower (95% CI)	Upper (95% CI)
Control	76.3	1.51	73.3	79.2
Group 1	85.8	1.13	83.5	88.0
Group 2	86.9	1.08	84.8	89.0
Group 3	86.0	1.12	83.8	88.2

Values represent estimated marginal means obtained from the Beta regression model with a logit link function.

Overall, the integrated analysis indicates that microbial culture supplementation was consistently associated with increased egg production throughout the experimental period, promoting improved productive performance relative to the control group. Although small differences were observed among supplemented groups, all administration frequencies resulted in superior performance compared with the control group, with the biweekly protocol being associated with the highest mean production values observed during the evaluation period.

### Intestinal microbiota analysis

The intestinal microbiota was characterized through 16S rRNA gene sequencing, allowing an integrated assessment of microbial diversity and community composition among experimental groups. Analyses were conducted using complementary ecological metrics, including alpha diversity, beta diversity, and differential abundance analysis.

Alpha diversity evaluated using the Shannon Index, Observed Features, Faith’s Phylogenetic Diversity, and Evenness, showed no statistically significant differences among experimental groups, indicating maintenance of microbial richness, phylogenetic diversity, and community uniformity across treatments. These findings suggest structural stability of the intestinal microbiota.

In contrast, beta diversity revealed significant differences in microbial community composition among groups, indicating structural reorganization of bacterial communities. Distance based metrics demonstrated consistent separation of Groups 1 and 2 from the control group, whereas Group 3 exhibited less pronounced differentiation, suggesting a reduced impact on microbial community structure.

Differential abundance analysis was performed using the ANCOM BC method, allowing the identification of specific alterations across different taxonomic levels.

Evaluation at the order level provided evidence of broader structural changes within the intestinal microbiota, highlighting shifts in major bacterial groups associated with the intestinal environment.

Group 1 exhibited enrichment of several bacterial orders, including *Mycobacteriales, Synergistales*, and UBA1381, accompanied by depletion of RF32, *Methanomassiliicoccales*, and *Acetivibrionales*, indicating strong modulation and structural reorganization of the microbial community (Fig. 3).

**Fig. 3:**
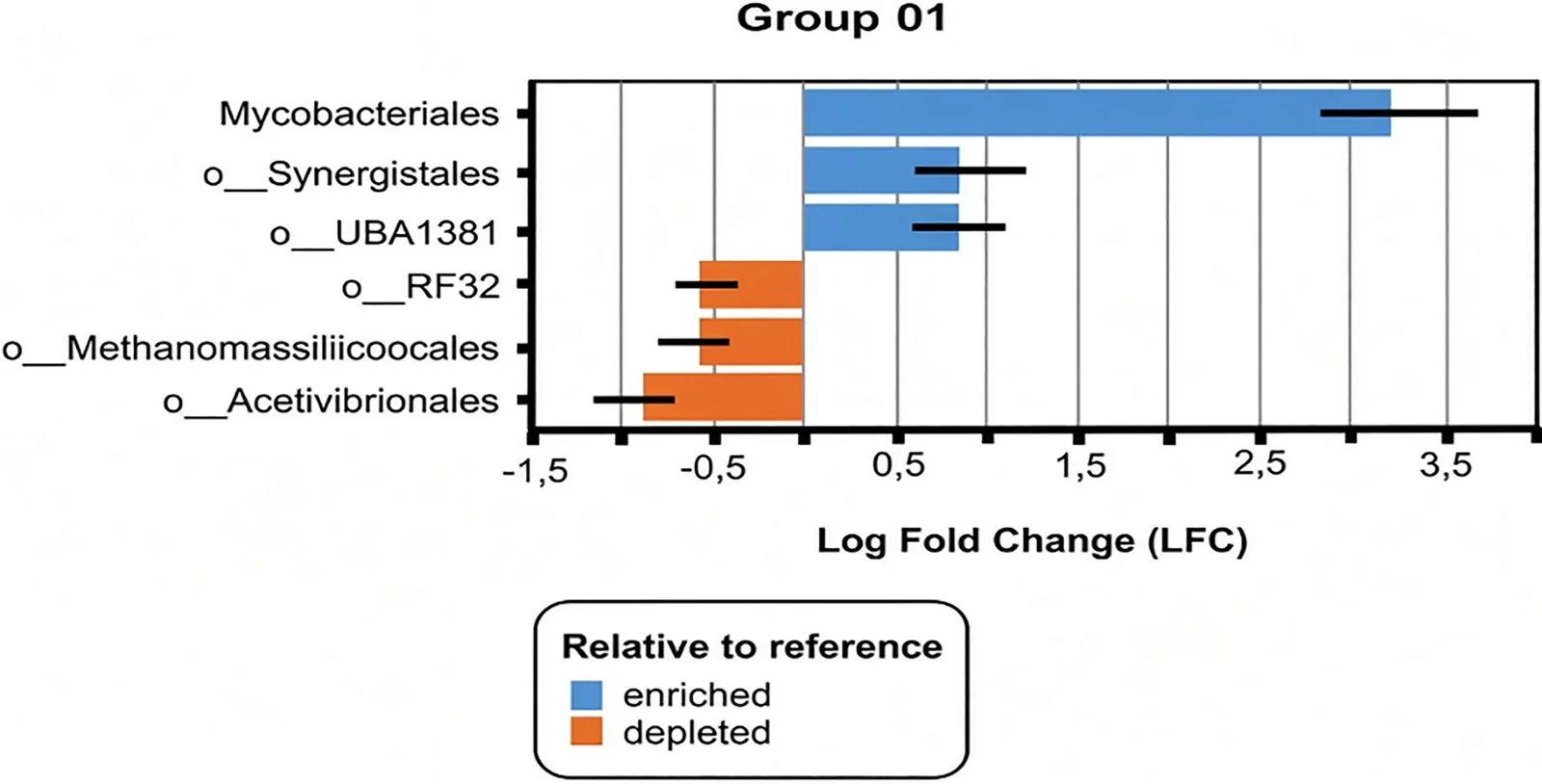
Differential abundance of bacterial taxa at the order level in Group 1 relative to the control group. Positive values indicate enriched taxa, whereas negative values indicate depleted taxa.

Group 2 exhibited a more specific pattern, characterized by depletion of *Clostridiales* and *Saccharimonadales*, indicating selective modulation of the intestinal microbiota (Fig. 4).

**Fig. 4:**
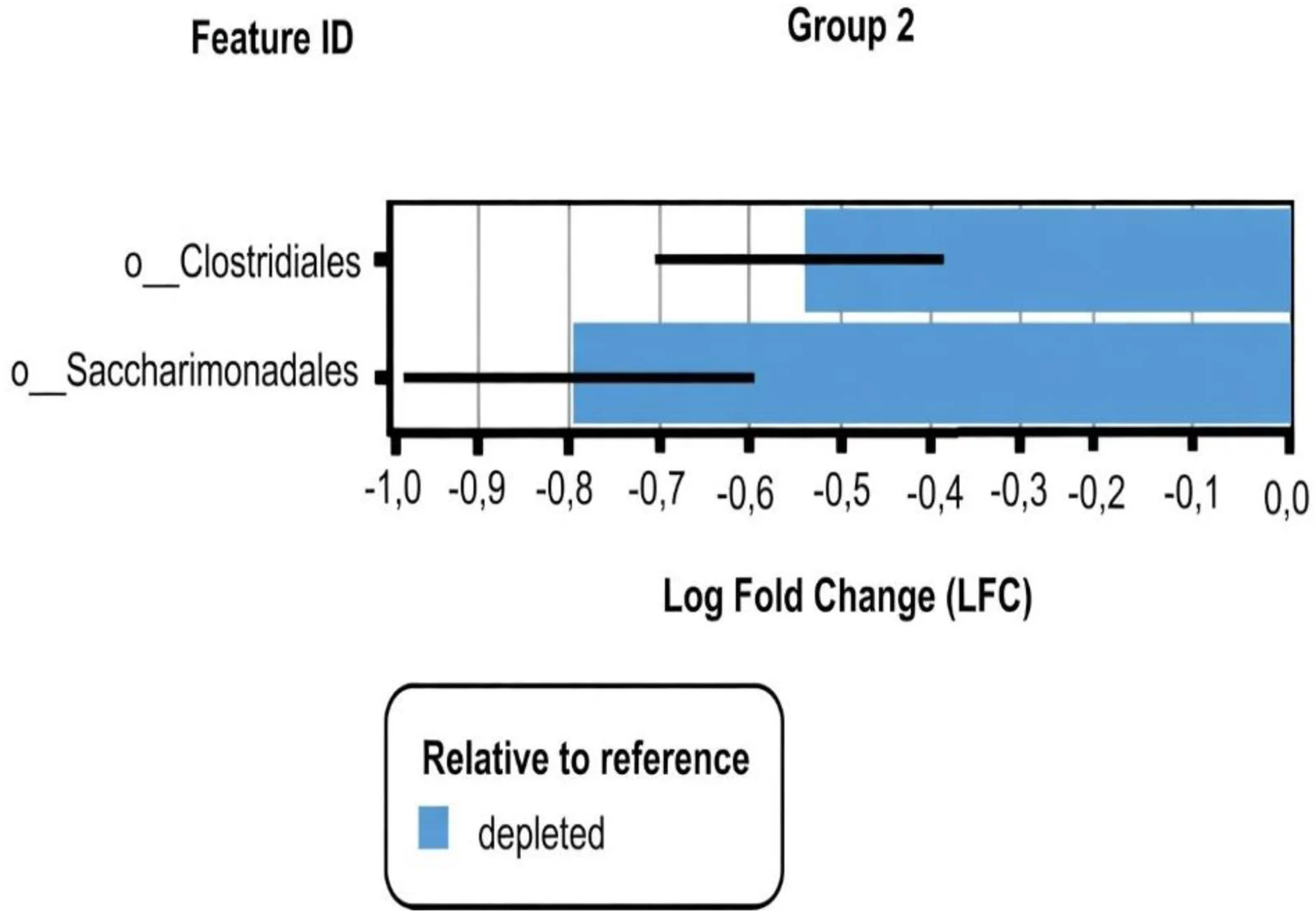
Differential abundance of bacterial taxa at the order level in Group 2 relative to the control group. Positive values indicate enriched taxa, whereas negative values indicate depleted taxa.

Group 3 exhibited a more heterogeneous response, characterized by enrichment of *Mycobacteriales* and *Actinomycetales* and marked depletion of *Rickettsiales*, indicating lower consistency and stability in microbial modulation (Fig. 5).

**Fig. 5:**
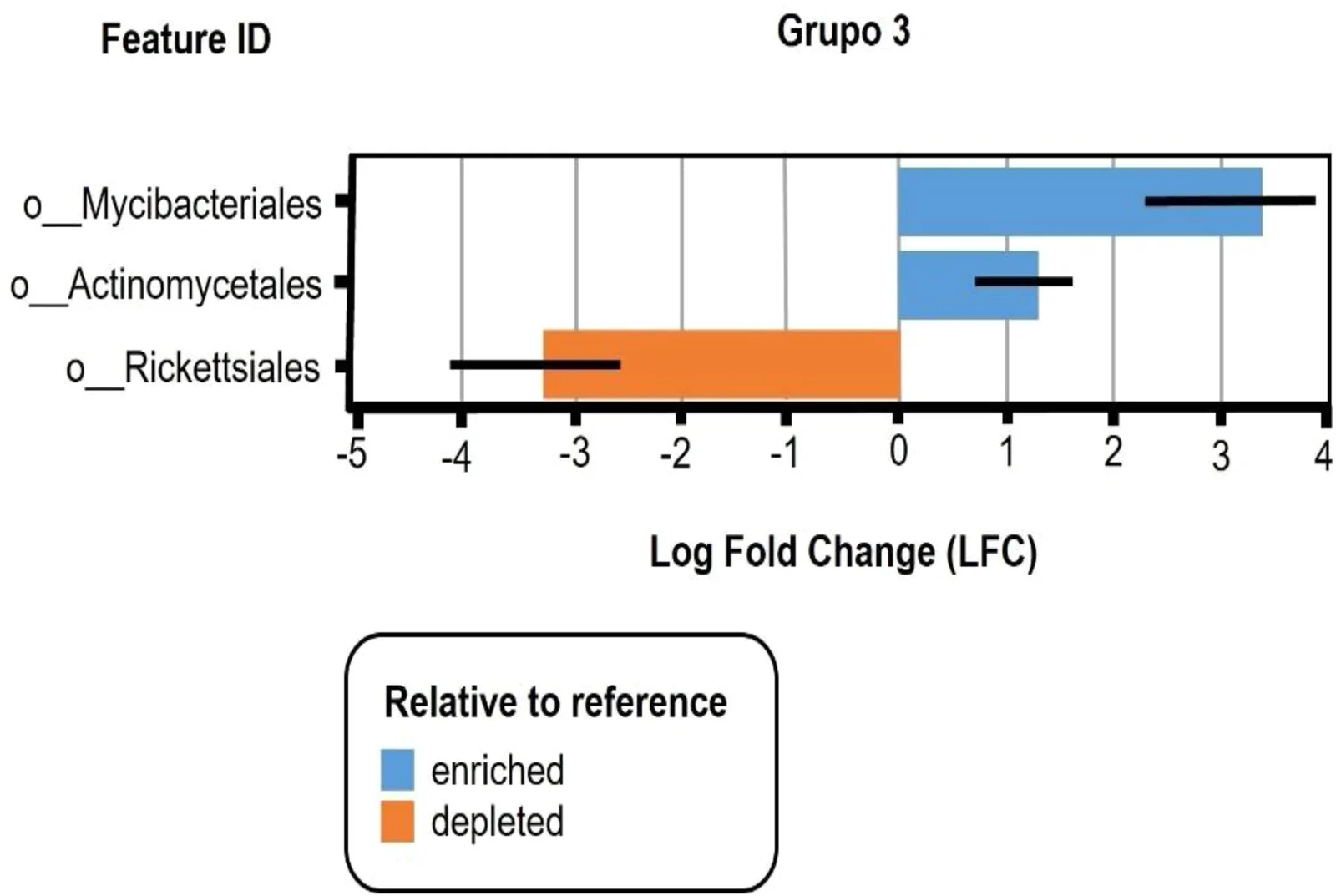
Differential abundance of bacterial taxa at the order level in Group 3 relative to the control group. Positive values indicate enriched taxa, whereas negative values indicate depleted taxa.

Analysis at the family level provided a more refined interpretation of microbial alterations, revealing more specific modulation patterns associated with the different administration protocols.

Group 1 exhibited enrichment of the families UBA020 and UBA1381, whereas taxa such as *Methanomethylophilaceae*, DSM 8532, and CAG 977 were depleted, indicating selective modulation of microbial community composition (Fig. 6).

**Fig. 6:**
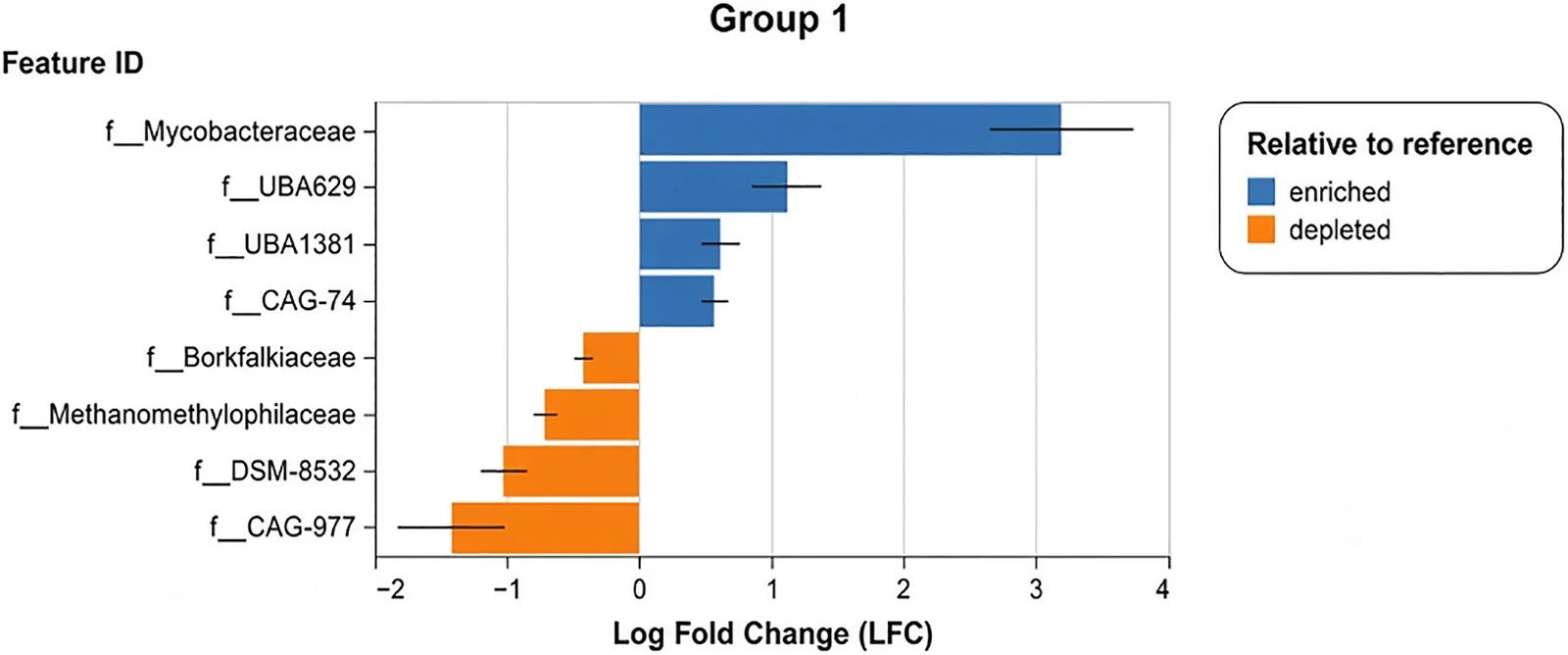
Differential abundance of bacterial taxa at the family level in Group 1 relative to the control group. Positive values indicate enriched taxa, whereas negative values indicate depleted taxa.

Group 2 exhibited consistent depletion of *Acutalibacteraceae, Methanomethylophilaceae, Clostridiaceae* 222000, DSM 8532, and *Nanoperiomorbaceae*, indicating a reduction in taxa potentially associated with intestinal metabolism (Fig. 7).

**Fig. 7:**
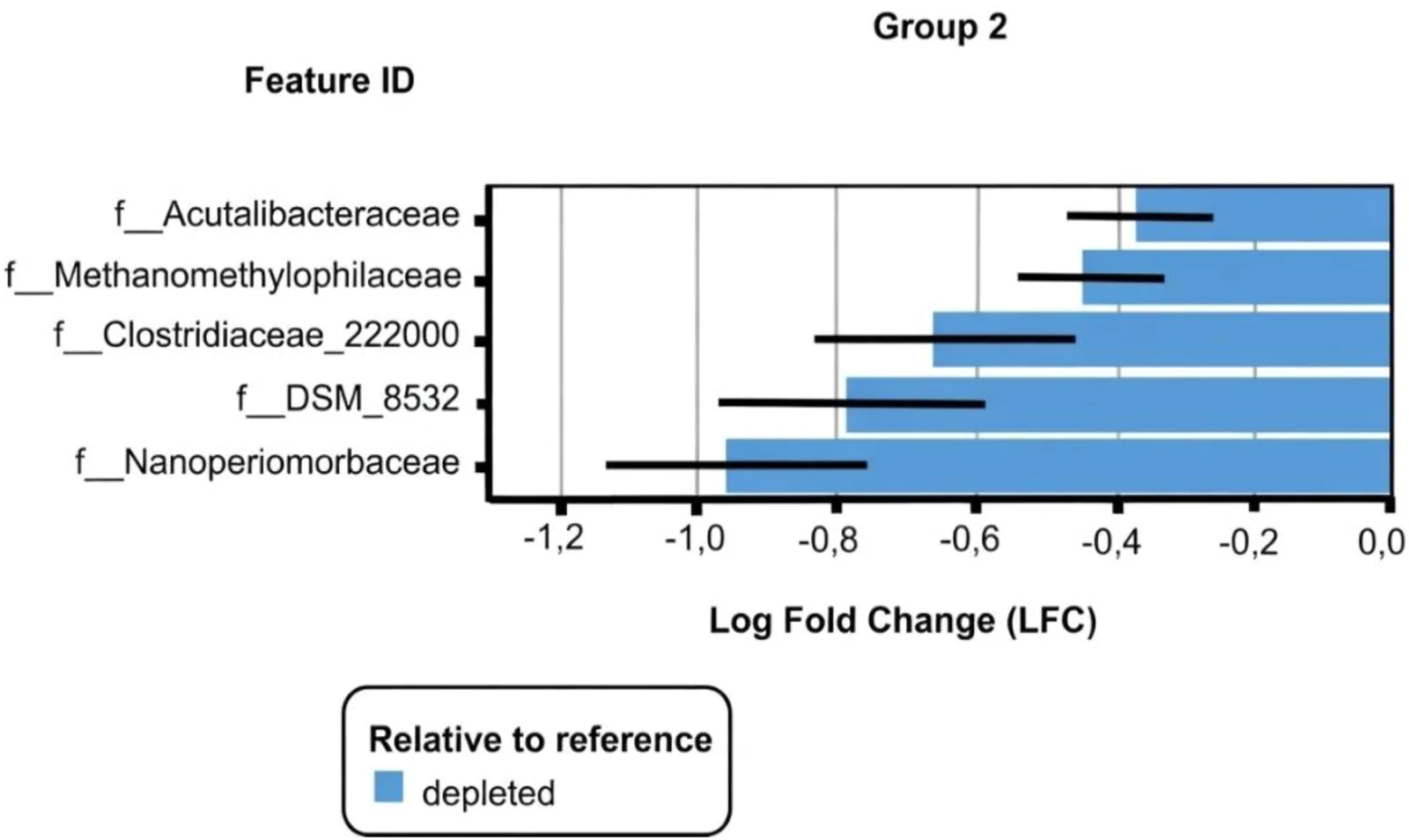
Differential abundance of bacterial taxa at the family level in Group 2 relative to the control group. Positive values indicate enriched taxa, whereas negative values indicate depleted taxa.

Group 3 maintained a variable pattern, characterized by enrichment of *Mycobacteriales* and *Actinomycetales* and depletion of *Rickettsiales*, indicating less consistent and less stable modulation of the intestinal microbiota compared with the other treatments (Fig. 8).

**Fig. 8:**
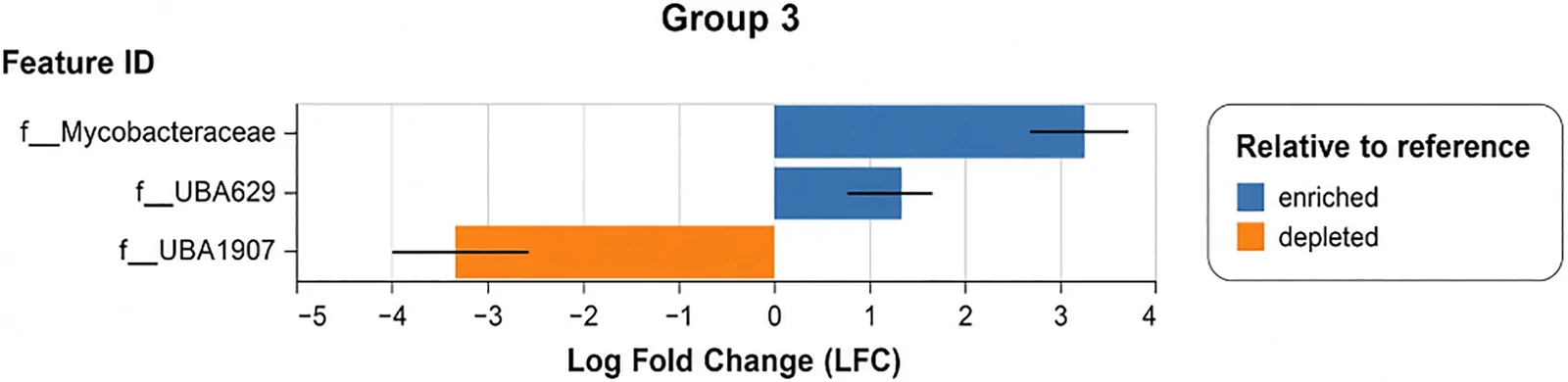
Differential abundance of bacterial taxa at the family level in Group 3 relative to the control group. Positive values indicate enriched taxa, whereas negative values indicate depleted taxa.

Analysis at a more detailed taxonomic level provided a functional interpretation of microbial alterations, highlighting potential metabolic and physiological implications.

Group 1 exhibited enrichment of Corynebacterium, WRAI01, CAG 41, and SFMI01, whereas taxa such as *Lachnoclostridium* B, *Massilistercora*, HGM16780, and CAG 977 were depleted, indicating functional reorganization of the intestinal microbiota (Fig. 9).

**Fig. 9:**
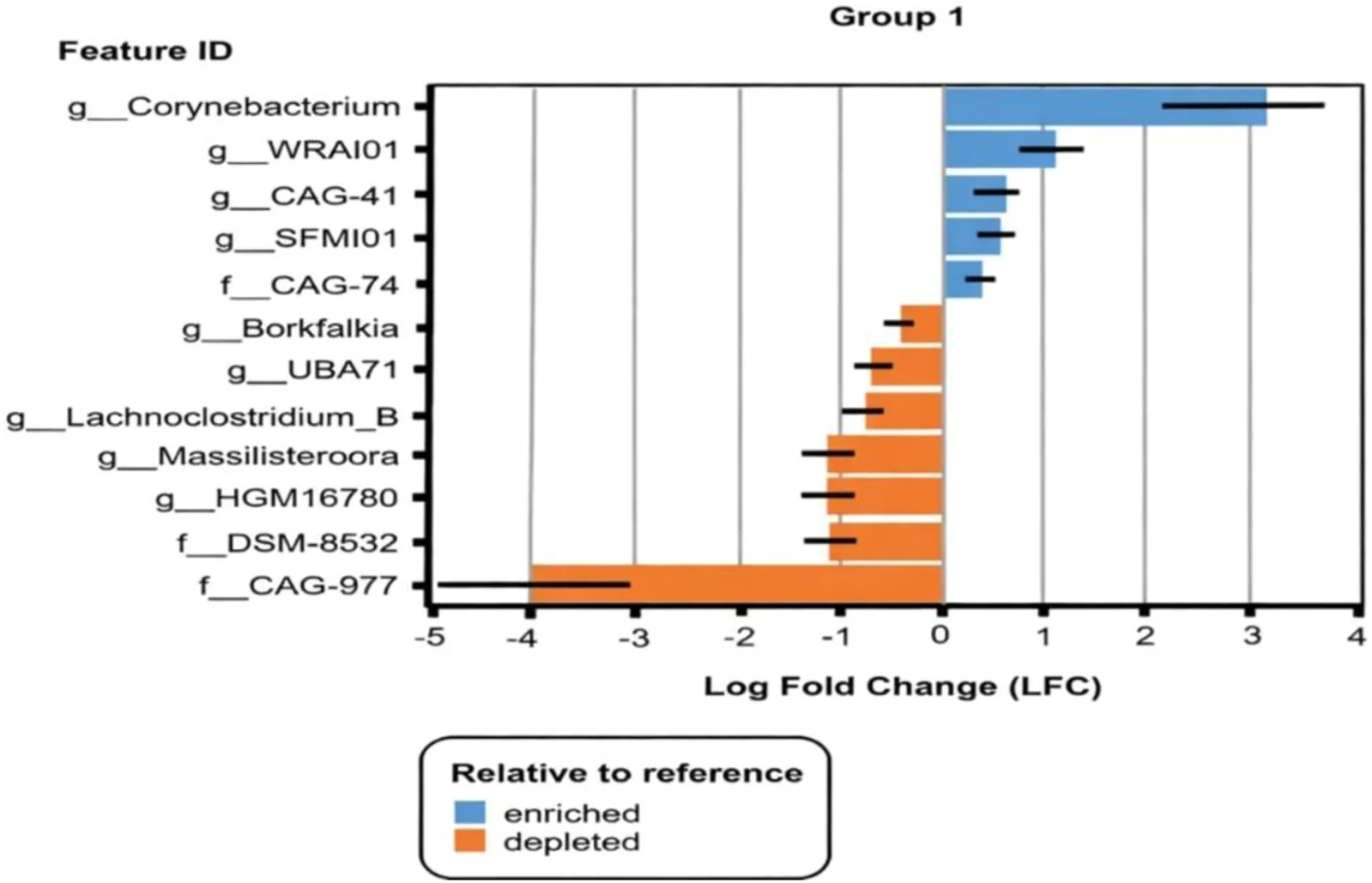
Differential abundance of bacterial taxa at the genus level in Group 1 is relative to the control group. Positive values indicate enriched taxa, whereas negative values indicate depleted taxa.

Group 2 exhibited a pattern characterized by depletion of multiple taxa, including *Lawsonibacter, Fimenecus, Massilistercora, Ruthenibacterium, Scatocola, Copromorpha*, QHMH01, and Zag11, together with specific enrichment of CAG 267, indicating selective modulation of the intestinal microbiota (Fig. 10).

**Fig. 10:**
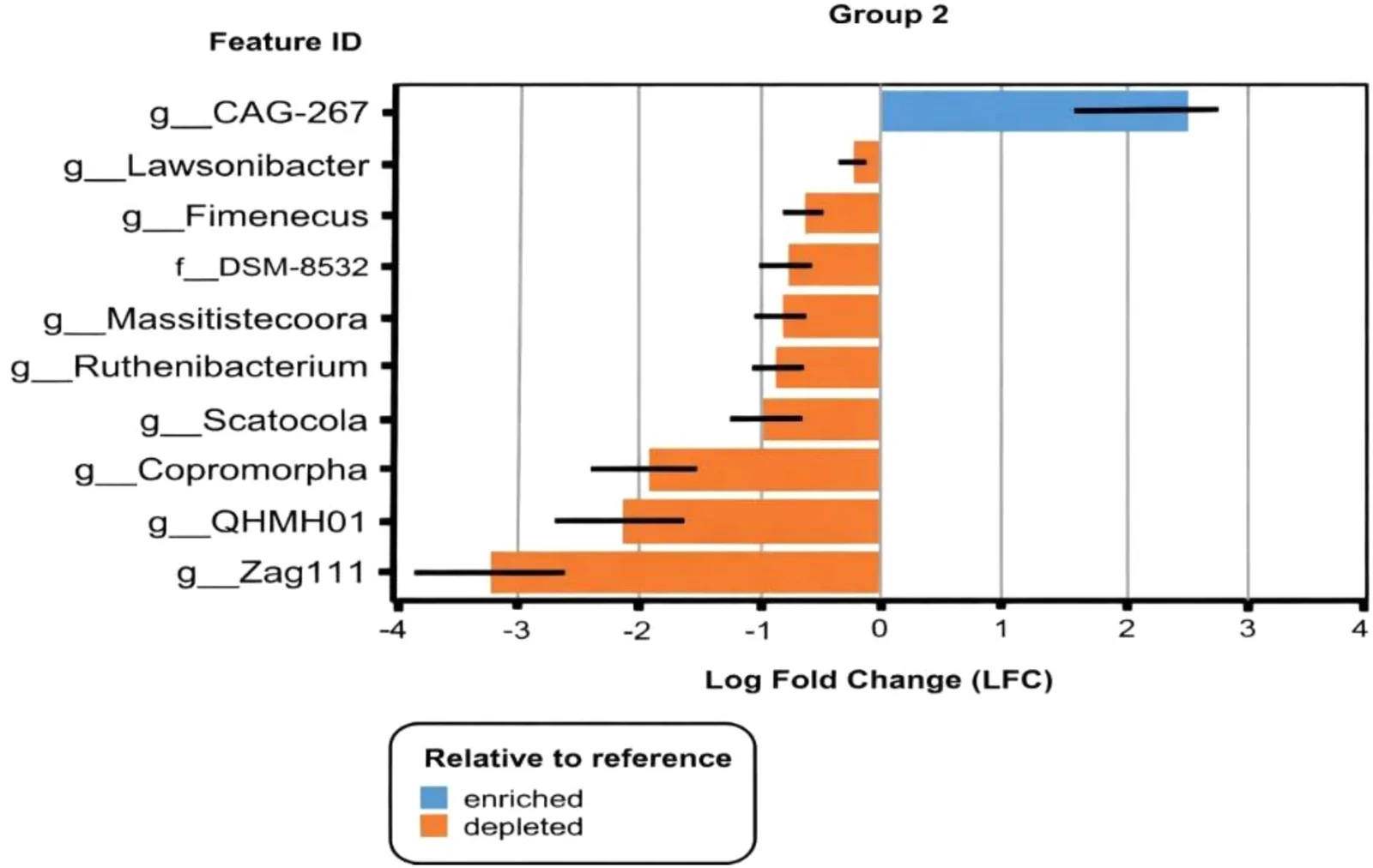
Differential abundance of bacterial taxa at the genus level in Group 2 relative to the control group. Positive values indicate enriched taxa, whereas negative values indicate depleted taxa.

Group 3 exhibited enrichment of *Corynebacterium*, QAKW01, and CAG 349, accompanied by depletion of UBA71, *Limenecus, Scatocola*, QHMH01, and UBA1997, maintaining a heterogeneous and less consistent response pattern (Fig. 11).

**Fig. 11:**
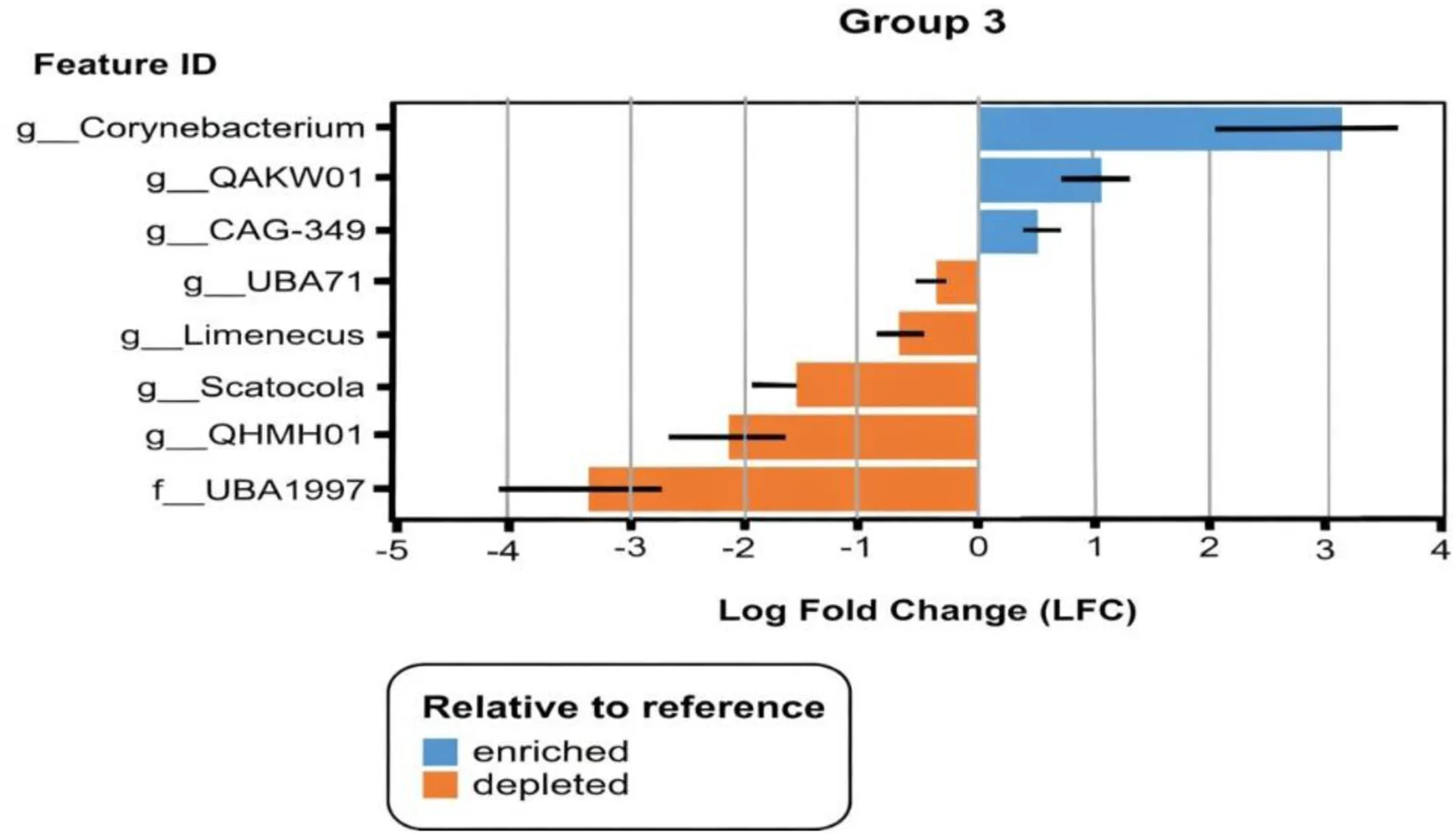
Differential abundance of bacterial taxa at the genus level in Group 3 relative to the control group. Positive values indicate enriched taxa, whereas negative values indicate depleted taxa.

### Intestinal clinical evaluation

#### Occurrence of intestinal gas

Evaluation of intestinal gas occurrence revealed a significant effect of age (χ² = 3.96; p = 0.047) and the quadratic age term (χ² = 4.03; p = 0.045), indicating a nonlinear pattern throughout the experimental period. Although the overall effect of treatment group showed only a trend toward statistical significance at the 5% level (χ² = 6.31; p = 0.097), analysis of exponentiated coefficients [Exp(B)] demonstrated that the occurrence rate of intestinal gas was approximately 2.13 times lower in Group 1 compared with the control group [Exp(B) = 0.469; p = 0.022], indicating a consistent improvement in intestinal stability under this experimental protocol.

Groups 2 and 3 also exhibited a numerical reduction in the occurrence of intestinal gas relative to the control group, although these differences were not individually significant. Overall, the results suggest that protocols involving more frequent microbial administration were associated with a lower occurrence of intestinal fermentative disturbances throughout the experimental period (Fig. 12).

**Fig. 12:**
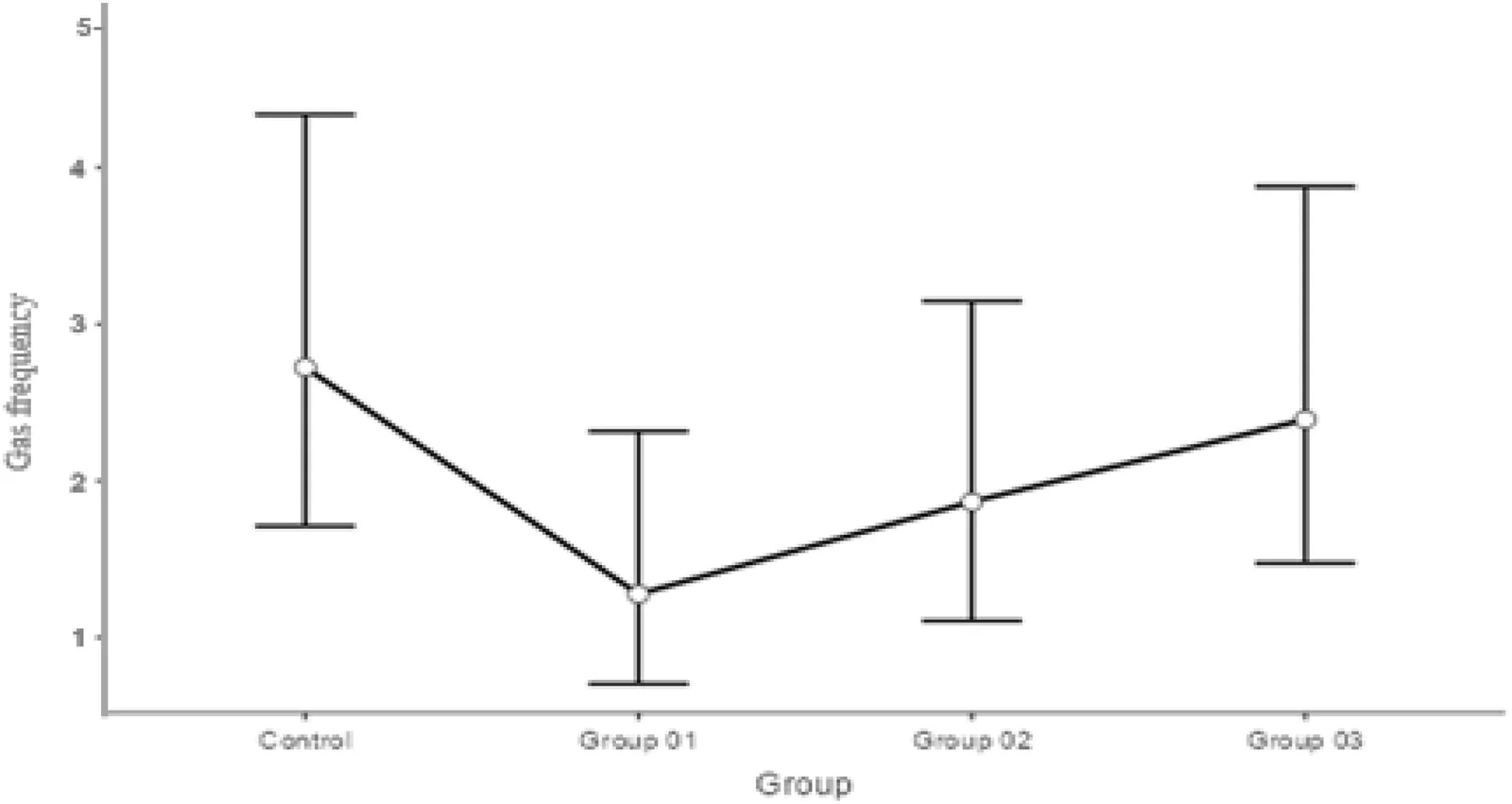
Occurrence of intestinal gas in broiler breeder hens subjected to different microbial administration protocols. Points represent estimated means obtained from the Generalized Linear Model with a Negative Binomial distribution and log link function, whereas error bars represent 95% confidence intervals.

#### Presence of kcus

Evaluation of intestinal mucus occurrence revealed a significant effect of treatment group (χ² = 7.95; p = 0.047) and age (χ² = 4.44; p = 0.035), indicating that microbial administration protocols influenced the occurrence of this clinical parameter throughout the experimental period.

Analysis of exponentiated coefficients [Exp(B)] demonstrated that the occurrence rate of intestinal mucus was approximately 2.58 times lower in Group 1 compared with the control group [Exp(B) = 0.388; p = 0.007], representing the most pronounced effect among the treatments evaluated. Groups 2 and 3 also exhibited a tendency toward reduced mucus occurrence, although these differences did not reach individual statistical significance.

Overall, the results indicate that microbial supplementation was associated with improved intestinal stability, particularly under protocols involving more frequent administration, reducing the occurrence of clinical alterations consistent with digestive disturbances (Fig. 13).

**Fig. 13:**
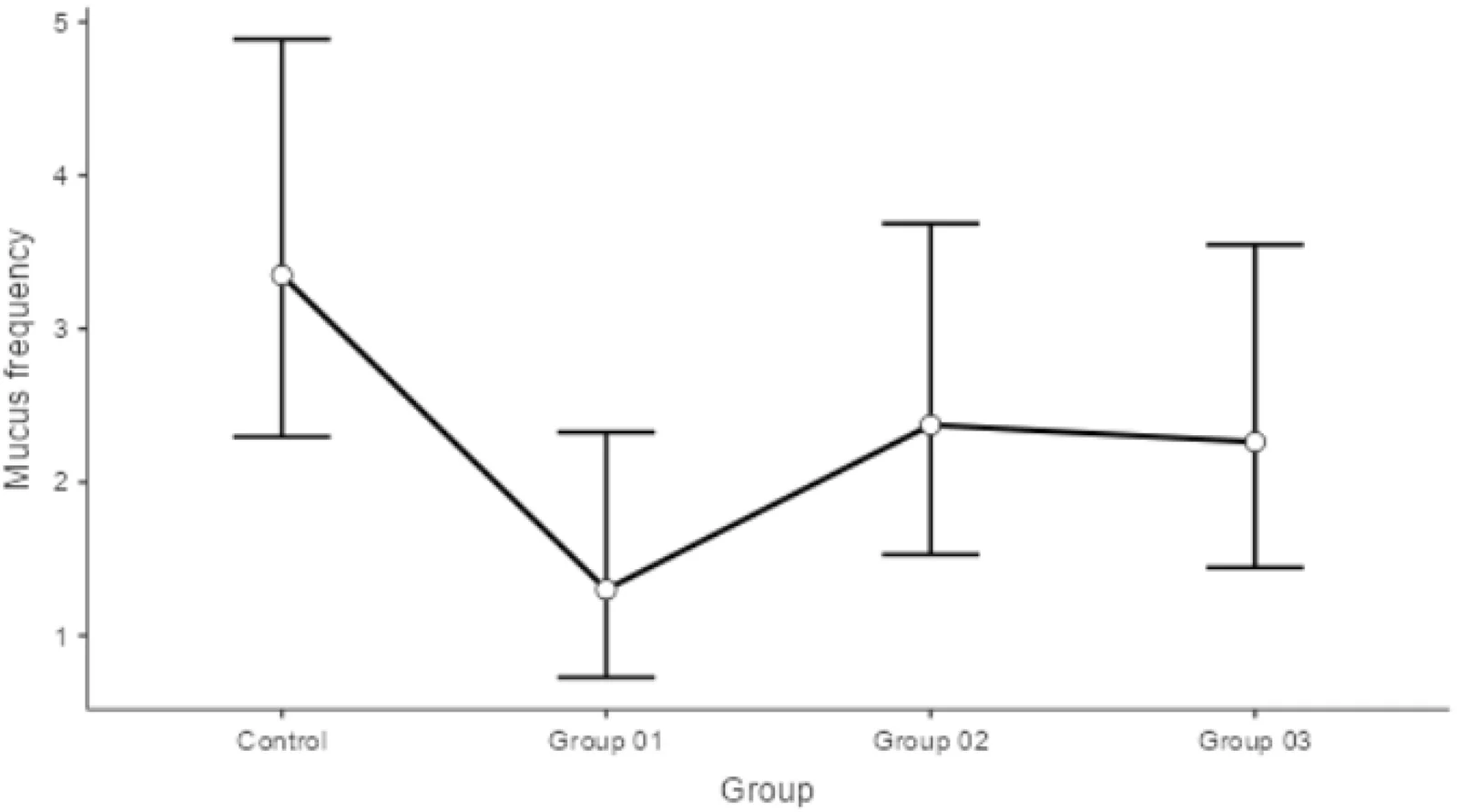
Occurrence of intestinal mucus in broiler breeder hens subjected to different microbial administration protocols. Points represent estimated means obtained from the Generalized Linear Model with a Negative Binomial distribution and log link function, whereas error bars represent 95% confidence intervals.

#### Relationships among intestinal microbiota, intestinal condition, and productive performance

Overall, the results demonstrate that microbial culture administration protocols promoted selective modulation of the intestinal microbiota, characterized by consistent alterations in taxonomic composition without significant effects on overall microbial diversity. This pattern indicates that the observed effects were predominantly associated with structural and functional reorganization of the microbiota rather than changes in microbial richness or evenness.

Groups 1 and 2 exhibited a greater capacity for microbial modulation, with consistent alterations across multiple taxonomic levels, indicating a more stable and targeted biological response. These findings were accompanied by improved intestinal stability and lower mortality rates throughout the experimental period. In contrast, Group 3 exhibited a less consistent and more variable pattern, suggesting that longer intervals between administrations may reduce the efficiency of microbial modulation over time, thereby limiting the capacity for bacterial community reorganization.

The observed alterations in microbiota composition were associated with improvements in intestinal clinical indicators, particularly reductions in events related to mucus occurrence and intestinal gas production, reflecting greater stability of the intestinal environment throughout the experimental period. The integrated analysis of intestinal clinical parameters further reinforced this pattern, suggesting that microbial modulation contributes to the maintenance of intestinal homeostasis and the functional balance of the gastrointestinal ecosystem.

Furthermore, the consistent association among microbiota modulation, improved intestinal condition, and productive performance reinforces the functional role of the microbiota as a central component in the physiological regulation of the gastrointestinal tract. In this context, the selective reorganization of microbial communities may be directly associated with optimization of digestive processes and improved nutrient utilization efficiency, resulting in reduced mortality and enhanced production throughout the experimental period.

#### Integration of intestinal clinical parameters

As a complementary approach, integration of intestinal clinical parameters allowed the assessment of the overall intestinal condition of birds throughout the experimental period.

The integrated analysis of intestinal clinical parameters through the Integrated Intestinal Condition Index revealed a significant effect of treatment group (χ² = 13.45; p = 0.004) and a significant quadratic effect of age (χ² = 4.97; p = 0.026), indicating consistent differences in intestinal stability among treatments throughout the experimental period.

Supplemented groups exhibited lower values of the Integrated Intestinal Condition Index compared with the control group, particularly Group 1 (−0.711), followed by Groups 2 (−0.317) and 3 (−0.249), indicating a lower integrated occurrence of intestinal clinical alterations and greater physiological stability of the gastrointestinal tract. The control group exhibited the highest mean value of the integrated index (0.289), reinforcing the greater overall occurrence of intestinal clinical alterations throughout the experimental period.

Additionally, these findings reinforce the results observed individually for parameters related to intestinal mucus and gas occurrence, indicating a favorable effect of microbial supplementation on the intestinal condition of the birds.

#### Evaluation of intestinal lesion scores

Evaluation of intestinal lesion scores revealed a significant effect of intestinal clinical classification (χ² = 96.72; p < 0.001), bird age (χ² = 67.46; p < 0.001), as well as significant interactions between clinical classification and age (χ² = 143.81; p < 0.001) and between intestinal clinical classification and microbial administration protocols (χ² = 32.75; p < 0.001), demonstrating that the dynamics of intestinal lesions varied according to bird age and the different microbial supplementation protocols.

Overall, supplemented groups exhibited higher estimated marginal means for the Normal category, indicating a higher expected frequency of intestines without macroscopic alterations compared with the control group. Group 1 presented the highest estimated value for this category (52.88), followed by Groups 2 (48.44) and 3 (45.82), whereas the control group exhibited the lowest estimated value (39.13). This pattern indicates that microbial supplementation protocols, particularly the most frequent administration schedule, favored the maintenance of intestinal integrity.

Furthermore, supplemented groups exhibited lower estimated marginal means for the more severe lesion categories, particularly Group 1. For Score 2, Group 1 presented an estimated value of (4.78), lower than that observed in the control group (6.61). For Score 3, representing the most severe category within the classification system used, Group 1 presented an estimated value of (3.44), whereas the control group exhibited a value of (9.73). This pattern suggests a reduction in the expected occurrence of more severe intestinal lesions in groups receiving microbial supplementation, with the most consistent response observed in Group 1.

The results observed for intestinal lesion scores reinforce the findings previously identified for clinical parameters related to intestinal gas occurrence, intestinal mucus occurrence, and integrated intestinal condition, demonstrating that microbial supplementation was associated not only with functional improvement of the intestinal environment but also with preservation of intestinal mucosal integrity throughout the experimental period (Fig. 14).

**Fig. 14:**
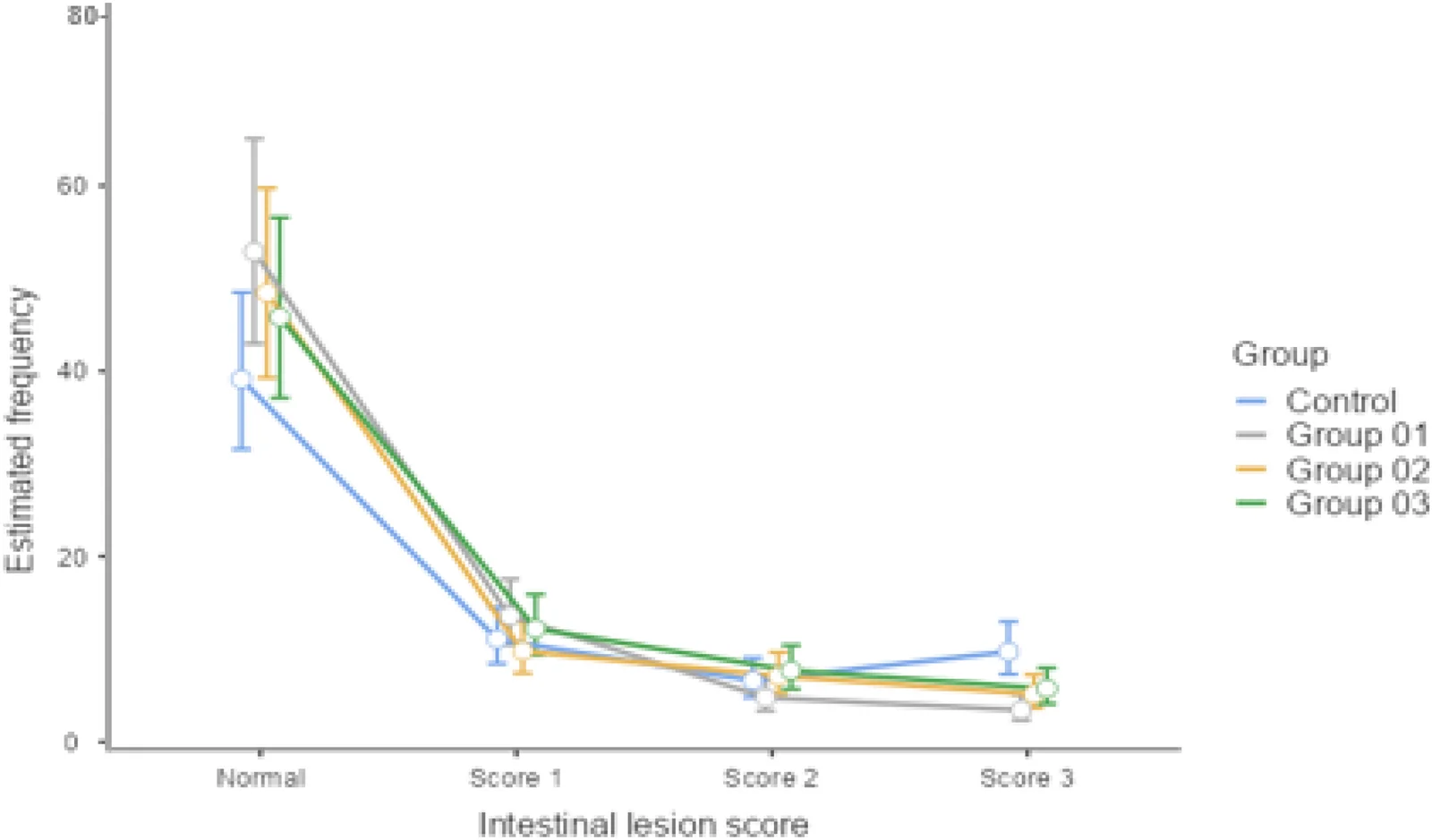
Distribution of intestinal lesion scores in broiler breeder hens subjected to different microbial administration protocols. Points represent estimated marginal means obtained from the Generalized Linear Model with a Negative Binomial distribution and log link function, whereas error bars represent 95% confidence intervals.

## Discussion

The results of this study demonstrate that the administration frequency of an undefined competitive exclusion culture (Normal Avian Gut Flora — NAGF) plays a determinant role in modulating cecal microbiota structure, gut integrity, and flock performance metrics in broiler breeder hens during the early production phase (24 to 32 weeks of age). By utilizing a longitudinal approach under commercial field conditions, this trial captured the continuous interactions among microbial succession, intestinal clinical health, and reproductive efficiency, reinforcing targeted microbial modulation as a practical strategy for flock health management (Yadav and Jha, 2019; Jha et al., 2020).

Flock mortality exhibited a characteristic nonlinear pattern over time, peaking at approximately 28 weeks of age before stabilizing. This trajectory aligns with established physiological dynamics in broiler breeders, where peak lay coincides with severe metabolic strain, hormonal reorganization, and heightened susceptibility to enteric dysbiosis (Romano, 2017). Under these field pressures, the biweekly protocol (Group 2) was associated with a lower estimated mortality rate relative to the unsupplemented Control group [Exp(B)=0.753; P=0.012]. Conversely, extending the administration window to 21 days (Group 3) was associated with a higher estimated mortality rate relative to the Control group [Exp(B)=1.258; P=0.042].

This adverse outcome observed in Group 3 warrants careful biological interpretation. The elevated mortality rate associated with the 21-day administration protocol highlights that infrequent microbial supplementation is not merely ineffective but may be biologically counterproductive during high-stress production phases. In broiler breeder hens at peak lay, the 21-day interval appears to create prolonged non-supplemented gaps wherein transient competitive exclusion pressure dissipates. This hypothesis is strongly supported by our beta diversity analyses, which demonstrated that Group 3 failed to maintain any statistical separation from the unsupplemented Control group across all distance matrices. Consequently, the rapid wash-out of beneficial culture strains during these extended 18-day unsupplemented intervals may trigger transient microbial instability, leaving the intestinal mucosa vulnerable to opportunistic colonization and metabolic disruption during peak production stress. These findings clearly demonstrate that the clinical and physiological benefits of undefined microbial cultures are strictly frequency-dependent, and infrequent administration protocols (such as 21 days) should be explicitly avoided in commercial health management programs. A key methodological consideration of the present study is the allocation of each

Egg production dynamics closely mirrored survival rates, with supplemented groups maintaining superior production consistency. Prior investigations established that effective intestinal microbiota modulation enhances lay performance and egg shell quality by optimizing nutrient digestibility, mucosal absorptive surface area, and energy partitioning (Abdelqader et al., 2013; Guo et al., 2017; Xu et al., 2023). In the present evaluation, the biweekly administration schedule achieved the highest laying persistence. This suggests that intermediate dosing frequencies provide the physiological stability necessary to meet the high metabolic turnover of early lay without over-stimulating mucosal immune responses (Dong et al., 2024).

The high throughput 16S rRNA gene sequencing data provides a clear mechanistic foundation for these observed performance trends. The absence of significant differences in alpha diversity metrics (Observed Features, Shannon Index, Faith’s PD, and Evenness) across treatments (P>0.05) indicates that NAGF supplementation did not disrupt the fundamental species richness or community uniformity of the native cecal ecosystem. This stability in alpha diversity is desirable in adult poultry, as drastic shifts in overall richness can signal ecological instability or acute dysbiosis (Aruwa et al., 2021; Chen et al., 2023).

In contrast, beta diversity analyses revealed profound, dose-frequency dependent restructuring of the bacterial community. Pairwise PERMANOVA evaluations across Bray–Curtis, Jaccard, unweighted UniFrac, and weighted UniFrac distance matrices confirmed that Groups 1 (weekly) and 2 (biweekly) significantly shifted the cecal microbial profile compared to the Control group (P<0.05). Crucially, Group 3 (21-day interval) showed no statistical separation from the unsupplemented controls across Bray–Curtis (P=0.090), Jaccard (P=0.199), unweighted UniFrac (P=0.098), or weighted UniFrac distances (P=0.307). This lack of divergence proves that a 21-day administration interval is insufficient to permanently colonize or modulate the mature cecal microbiota, allowing the community to revert to an unsupplemented steady-state. These findings validate previous observations that competitive exclusion microorganisms require repeated inoculation within specific physiological windows to maintain colonization resistance against transient dysbiosis (Giannenas et al., 2012; Al Khalaifa et al., 2019).

Differential abundance analysis via ANCOM-BC revealed targeted taxonomic shifts across Order (level 4), Family (level 5), and Genus (level 6) that explain these health benefits. At the order level, Groups 1 and 3 exhibited enrichments in Mycobacteriales, whereas Group 2 was characterized by a reduction in Clostridiales and Saccharimonadales relative to controls. At the genus level, weekly supplementation (Group 1) promoted the enrichment of Corynebacterium while depleting Massilistercora and Lachnoclostridium. Biweekly supplementation (Group 2) significantly enriched unclassified CAG-267 while depleting Lawsonibacter, Massilistercora, and Copromorpha. The depletion of taxa such as Massilistercora and specific clostridial lineages in treated birds suggests a selective suppression of potential opportunistic gut fermenters that are frequently elevated during subclinical inflammatory events (Hoepers et al., 2024).

Complementing the metagenomic findings, macroscopic clinical health evaluations demonstrated a marked reduction in enteric pathology in supplemented flocks. Treated groups displayed a significantly lower incidence of intestinal gas accumulation, mucosal edema, and lower ordinal lesion scores, translating into an improved integrated intestinal condition index (P<0.01). These clinical observations agree with evidence showing that undefined competitive exclusion cultures reinforce tight junction integrity, stimulate mucin secretion, and suppress gas-producing anaerobic putrefaction (Krysiak et al., 2021; Yaqoob et al., 2022).

A key methodological consideration of the present study is the allocation of each administration protocol to a single commercial house. In large-scale field trials with broiler breeders, the house constitutes the natural experimental unit because birds within the same facility share identical environmental, management, and housing conditions. Replicating each treatment across multiple independent houses would have required a substantially larger number of synchronized flocks and was logistically unfeasible under commercial constraints. To mitigate potential pseudoreplication, performance and clinical data were analyzed using Generalized Linear Mixed Models with repeated measures over time and random effects for house, while microbiome analyses were conducted on independent biological pools (n=5 per group). Although this design limits the ability to fully separate treatment effects from possible house-related confounding factors, it provides high external validity by reflecting real-world commercial production conditions with 48,000 birds.

Synthesizing these multi-dimensional results reveals an integrated biological mechanism: regular administration of NAGF drives targeted bacterial shifts (beta diversity separation and selective taxon enrichment) without compromising overall community richness. This structural shift correlates with reduced subclinical intestinal inflammation and lower lesion scores, which in turn optimizes energy utilization, and is associated with a lower estimated mortality rate in Group 2. Under commercial production standards where antimicrobial growth promoters and prophylactic treatments are increasingly restricted (EC, 2003; Pessôa et al., 2012), establishing that a biweekly (15-day) application protocol yields optimal biological and survival efficiency offers a highly cost-effective, sustainable gut health strategy for commercial broiler breeder operations, whereas extended 21-day intervals should be avoided due to lack of efficacy and potential mortality rate.

## Data availability statement

The data supporting the findings of this study are available from the corresponding author upon reasonable request.

## Ethics declaration

This study was conducted in accordance with the ARRIVE (Animal Research: Reporting of In Vivo Experiments) guidelines.This study was approved by the Animal Use (CEUA) of the School of Veterinary Medicine and Animal Science (FMVZ), UNESP, Botucatu, SP, Brazil.(Certificate No. 23/2023)

## Declaration of generative AI and AI-assisted technologies in the manuscript preparation process

During the preparation of this work, the authors used ChatGPT (OpenAI) to assist with language translation, grammatical editing, and improvement of the manuscript's structure and readability. The authors reviewed, revised, and validated all generated content and take full responsibility for the accuracy, integrity, and content of the published article.

## Funding

This research did not receive any specific grant from funding agencies in the public, commercial, or not for profit sectors.

## Author Contribution

Dauísia Corsino de Souza: Conducted the experimental study, including the field activities, experimental procedures, sample collection, and data collection. Contributed substantially to the development of the study and manuscript preparation. Participated in the critical revision of the manuscript, approved the final version, and accepts responsibility for all aspects of the work. Roberto Carlos de Freitas: Contributed substantially to the statistical analysis of the experimental data, including statistical modeling and analysis of the results. Contributed to the writing and interpretation of the statistical results and critically revised the manuscript. Approved the final version and accepts responsibility for all aspects of the work. Manoel Garcia Neto: Contributed substantially to the statistical analysis, including the selection, application, and evaluation of statistical models, as well as the interpretation of the results. Contributed to the correction and critical revision of the manuscript. Approved the final version and accepts responsibility for all aspects of the work. Jumara Coelho Ticiano: Contributed substantially to data collection and data organization. Contributed to the writing and critical revision of the manuscript. Approved the final version and accepts responsibility for all aspects of the work. Raphael Lucio Andreatti: Contributed substantially to the experimental design and scientific development of the study. Provided critical scientific input and participated in multiple rounds of critical revision of the manuscript. Approved the final version and accepts responsibility for all aspects of the work. Adriano Sakai Okamoto: Contributed substantially to the experimental design and scientific development of the study. Supervised and followed the project throughout its development and contributed to the critical revision of the manuscript. Approved the final version and accepts responsibility for all aspects of the work.

## Disclosures

The authors declare that they have no known competing financial interests or personal relationships that could have appeared to influence the work reported in this paper.
